# Analysis of the status quo of pelvic floor muscle and the effect of pelvic floor muscle training in second pregnant women

**DOI:** 10.1097/MD.0000000000031370

**Published:** 2022-11-11

**Authors:** Jun Chao Zeng, Yu Ying Yang, Yan Shen

**Affiliations:** a Health Management Center, Union Hospital, Tongji Medical College, Huazhong University of Science and Technology, Wuhan, China; b Obstetrics and Gynecology Department, Tongji Hospital, Tongji Medical College, Huazhong University of Science and Technology, Wuhan, China.

**Keywords:** electrical stimulation combined with biofeedback, Kegel exercise, pelvic floor muscle function, second trimester

## Abstract

This study aims to investigate the current situation of pelvic floor muscle (PFM) in the second trimester postpartum 42 days by detecting the electrophysiological indexes of PFM on pelvic floor rehabilitation after 2 months.

**Methods:**

In total, 198 cases of second child puerpera were selected, who were delivered in our hospital between June 1, 2020, and December 10, 2020, and underwent outpatient reexamination 42 days after delivery.

**Results:**

Except for the rest post-baseline stage values, the pelvic floor surface electromyography evaluation values significantly differed from each other at the considered time points in group A, *P* < .05; on day 72 in group B, no obvious improvement in the evaluation values was observed as than those on day 42 (*P* > .05) except for the endurance contractions stage values. However, on day 102, all values were markedly different at each considered time point (*P *< .05). On day 102 postpartum, the evaluation values of group A in the rest pre-baseline stage, the time before and after peak of phasic (flick) contractions stage, and the endurance contractions stage were significantly improved to those in group B with all *P *< .05. On day 42 after parturition, each index of the tonic contractions stage was higher after spontaneous labor than that after cesarean; the differences were all significant, *P *< .05, but on day 102 postpartum, all of the values exhibited no difference between the 2 modes. In only the phasic (flick) contractions stage at 42th, were the values of younger mothers obviously higher, *P *= .025; the other stage values for different ages of women during different time periods were not statistically significant, *P* > .05.

**Conclusions:**

In the short term, the effect of biofeedback plus electrical stimulation on the PFM function in second pregnant women was better than that of the Kegel exercise, but with time, there was no significant difference between the 2 training methods on the recovery of the PFM.

## 1. Introduction

Pelvic floor muscle (PFM) is composed of the levator ani muscle and the caudal muscle, which is very important for supporting pelvic organs. It can also synergize with the bladder, rectum, and sexual function.^[1]^ Pelvic floor dysfunction (PFD) is a pathological change in the pelvic floor structure resulting in an abnormal function of the corresponding organs. It is a common disease that affects the lives of many women globally and seriously threatens their physical and mental health and quality of life.^[2,3]^

In a woman’s life, there is approximately a 50% chance of suffering from pop, 11% to 19% chance of undergoing surgery due to prolapse or urinary incontinence,^[4]^ and 11.1% life-long risk of having pelvic organ prolapse (POP) or urinary incontinence surgery at the age of 80 years. In the United States, approximately 200,000 prolapsed patients receive surgery yearly, and the demand for medical services related to prolapse is expected to double with the population,^[5]^ but only 25% seek or receive treatment.^[6]^ In China, the incidence rate has increased yearly with the acceleration of population aging and the opening of the 2-child policy. PFD has developed into 1 of the 5 common chronic diseases, and the risk of recurrence is as high as 30%.^[7,8]^

Pregnancy and childbirth are the main factors causing PFD.^[9]^ The pathogenesis of postpartum PFD is mainly manifested in the relaxation of the pelvic floor-supporting tissue.^[10]^ The higher the number of childbirth times, the higher the frequency and severity of PFM damage.^[11]^ Therefore, early postpartum PFM rehabilitation exercise is particularly important; it cannot only promote the recovery of physical function after delivery but also improve the long-term pelvic floor condition.^[12]^ British guidelines recommend PFM exercise during the first pregnancy to prevent the occurrence of urinary incontinence.^[13]^ Chinese guidelines for the diagnosis and treatment of POP in 2020 also pointed out that PFM exercise can enhance PFM contractility and reduce the incidence of vaginal and uterine prolapse.^[14]^ However, previous studies did not use objective tests (such as the pad test) to describe the complete effectiveness of PFM training.^[15]^

This study aimed to understand the PFM status in women with a second child 42 days after delivery through pelvic floor screening. The participants were asked to carry out the Kegel exercise and the biofeedback plus electrical stimulation treatment for these women, and then the efficacy of the 2 training methods was compared to provide a theoretical basis for the selection of postpartum rehabilitation training methods.

## 2. Methods

### 2.1. Clinical data

This study was a retrospective analysis. In this retrospective case-control study, 198 cases of second-birth parturients who underwent a 42-day postnatal examination in a hospital in Wuhan from June 1, 2020, to December 10, 2020, were screened, including 100 cases of cesarean section and 98 cases of spontaneous labor. They were divided into 2 groups: group A was the biofeedback plus electrical stimulation group of 105 subjects with an average age of 32.20 ± 4.30 years, and group B was the Kegel training group of 93 subjects with an average age of 33.19 ± 3.97 years. The following information was recorded: maternal age, height, weight, body mass index (BMI), times of pregnancy, mode of delivery, weight gain during pregnancy, and neonate weight. These characteristics displayed no difference between the 2 groups. Both groups were asked to undergo PFM rehabilitation training for 2 months, starting from 42 days after delivery. General postpartum education was also provided to both groups at the time of discharge. All women were followed up at 42, 72, and 102 days after delivery.

The inclusion criteria were as follows: second child with single and full-term delivery and age ranging between 20 and 40 years. The exclusion criteria were as follows: acute inflammation of genitourinary system; history of chronic cough, chronic constipation, and pelvic surgery; history of diabetes, hypertension, and malignant tumor during pregnancy; family history of POP and urinary incontinence; natural delivery with a history of prolonged second stage of labor, episiotomy and instrumental midwifery; and cesarean section with a history of vaginal trial delivery.

### 2.2. Experimental methods

Group A: A biological and stimulation feedback instrument (Nanjing Medlander MLD B6T) was used for stimulating and contracting the PFMs. Surface electromyography (EMG), according to Glazer Protocol,^[16]^ was used to convert the EMG signal of the muscle reaction into standardized parameters, and the results were recorded concurrently.

One-on-one health education was provided by the medical staff to popularize the anatomy of the pelvic floor, the importance of muscle training, and the method to carry out the Kegel training. The correct method was as follows: lie on the back, fully relax the abdominal muscles, consciously contract the buttock muscles, and lift the anus upward, holding for 3 seconds with an interval of 5 to 10 seconds, repeat for 15 to 30 minutes for each group and 2 to 3 groups daily or 150 to 200 times a day.

### 2.3. Statistical analysis

SPSS 19.0 was used to analyze the data, and all results were expressed as *x* ± *s*. The counting data were expressed in terms of rate; *P* < .05 was considered statistically significant, and *P* < .01 revealed a dramatically significant difference. A *t* test was used to compare the general and inter-group test data of subjects. A 1-way analysis of variance (ANOVA) was used to compare the groups.

### 2.4. Ethical statement

The study was approved ruled that no formal consent was necessary by Medical Ethics Committee of Tongji Medical College, Huazhong University of Science and Technology. No administrative permission is required to access and use the medical records described in this study.

## 3. Results

### 3.1. Analysis of the pelvic floor function in the 2 groups

The results of the Glazer evaluation of the puerperal pelvic floor surface EMG of the 2 groups are presented in Table 1. No differences were observed in both indicators between the 2 groups, with all *P* values > .05.

**Table 1:** Comparison of the each value of pelvic floor sEMG assessed by Glazer at 42 days postpartum.

	Group A (n = 105)	Group B (n = 93)	*P*
Rest pre-baseline			
Average mean amplitude (uV)	9.81 ± 15.62	8.30 ± 10.06	.428
Mean amplitude variability (%)	0.25 ± 0.17	0.22 ± 0.19	.264
Phasic (flick) contractions			
Average peak amplitude (uV)	35.68 ± 17.30	32.10 ± 15.11	.125
Time Before peak (s)	0.54 ± 0.32	0.52 ± 0.32	.599
Time after peak (s)	0.71 ± 0.63	0.70 ± 0.59	.928
Tonic contractions			
Average peak amplitude (uV)	40.34 ± 19.85	36.57 ± 17.65	.161
Average mean amplitude (uV)	24.00 ± 12.79	21.85 ± 12.05	.227
Mean amplitude variability (%)	0.27 ± 0.12	0.26 ± 0.12	.755
Endurance contractions			
Average mean amplitude (uV)	20.64 ± 12.21	19.17 ± 10.93	.378
Mean amplitude variability (%)	0.23 ± 0.08	0.23 ± 0.09	.858
Rest Post-baseline			
Average mean amplitude (uV)	4.00 ± 3.47	4.75 ± 3.41	.127
Mean amplitude variability (%)	0.25 ± 0.17	0.24 ± 0.21	.650

*Group A: Biofeedback plus electrical stimulation; Group B: Kegel exercise.

### 3.2. Comparison of the scores of the pelvic floor function in each stage in group A after 2 months of treatment

**Figure 1.**
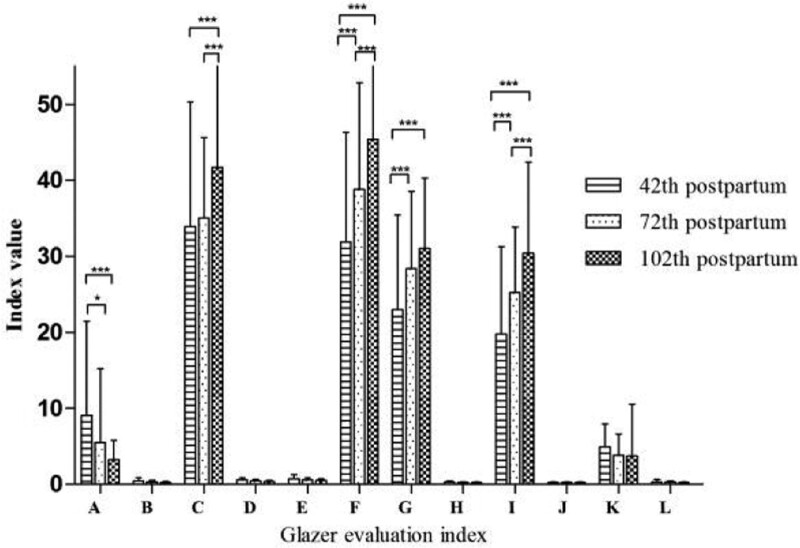
Pairwise comparison among 3 time points in group A. (A) Average mean amplitude (Rest pre-baseline); (B) Mean amplitude variability (Rest pre-baseline); (C) Average peak amplitude (Phasic (flick) contractions); (D) Time before peak (Phasic (flick) contractions); (E) Time after peak (Phasic (flick) contractions); (F) Average peak amplitude (Tonic contractions); (G) Average mean amplitude (Tonic contractions); (H) Mean amplitude variability (Tonic contractions); (I) Average mean amplitude (Endurance contractions); (J) Mean amplitude variability (Endurance contractions); (K) Average mean amplitude (Rest post-baseline); (L) Mean amplitude variability (Rest post-baseline).

In group A, the PFM function was reexamined after treatment. Except for the rest post-baseline stage values, the assessment values significantly differed from each other at all considered time points, *P* < .05, as illustrated in Table 2. A pairwise comparison (Fig. 1) revealed that the difference in the evaluation value between day 42 and 102 after childbirth in the rest post-baseline stage was not inapparent, *P* > .05. However, on day 102, the assessment value dropped to the normal range.

**Table 2:** Comparison among 3 time points in group A.

	42th	72th	102th	*P*
Rest pre-baseline				
Average mean amplitude (uV)	9.58 ± 14.15	4.79 ± 7.14	2.72 ± 2.08	.000
Mean amplitude variability (%)	0.41 ± 0.53	0.25 ± 0.17	0.20 ± 0.11	.000
Phasic (flick) contractions				
Average peak amplitude (uV)	35.68 ± 17.30	36.27 ± 10.68	42.14 ± 15.51	.002
Time before peak (s)	0.54 ± 0.32	0.40 ± 0.19	0.30 ± 0.87	.000
Time after peak (s)	0.71 ± 0.63	0.50 ± 0.36	0.44 ± 0.13	.000
Tonic contractions				
Average peak amplitude (uV)	28.04 ± 9.47	38.18 ± 12.80	45.65 ± 12.35	.000
Average mean amplitude (uV)	24.15 ± 12.81	29.63 ± 12.61	32.18 ± 11.00	.000
Mean amplitude variability (%)	0.27 ± 0.12	0.20 ± 0.06	0.19 ± 0.05	.000
Endurance contractions				
Average mean amplitude (uV)	20.64 ± 12.21	24.79 ± 8.56	28.78 ± 13.01	.000
Mean amplitude variability (%)	0.23 ± 0.08	0.22 ± 0.08	0.20 ± 0.06	.051
Rest Post-baseline				
Average mean amplitude (uV)	5.00 ± 2.65	3.40 ± 1.83	3.89 ± 9.12	.103
Mean amplitude variability (%)	0.28 ± 0.40	0.25 ± 0.17	0.20 ± 0.09	.078

### 3.3. Comparison of the scores of the pelvic floor function in each stage in group B after 2 months of treatment

**Figure 2.**
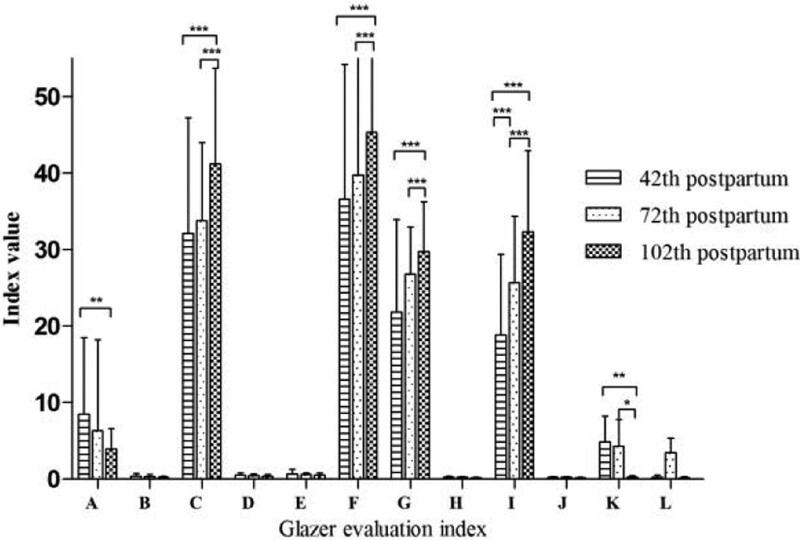
Pairwise comparison among 3 time points in group B. (A) Average mean amplitude (Rest pre-baseline); (B) Mean amplitude variability (Rest pre-baseline); (C) Average peak amplitude (Phasic (flick) contractions); (D) Time before peak (Phasic (flick) contractions); (E) Time after peak (Phasic (flick) contractions); (F) Average peak amplitude (Tonic contractions); (G) Average mean amplitude (Tonic contractions); (H) Mean amplitude variability (Tonic contractions); (I) Average mean amplitude (Endurance contractions); (J) Mean amplitude variability (Endurance contractions); (K) Average mean amplitude (Rest post-baseline); (L) Mean amplitude variability (Rest post-baseline).

After the Kegel exercise for 1 month, although the indexes had improved, no obvious significance was observed (*P* > .05, Fig. 2), except in the case of the endurance contractions stage values. However, on day 102, all values were markedly different at each time point (*P *< .05, Table 3).

**Table 3:** Comparison among 3 time points in group B.

	42th	72th	102th	*P*
Rest pre-baseline				
Average mean amplitude (uV)	8.50 ± 9.99	6.31 ± 11.91	3.93 ± 2.70	.003
Mean amplitude variability (%)	0.38 ± 0.37	0.32 ± 0.29	0.22 ± 0.19	.001
Phasic (flick) contractions				
Average peak amplitude (uV)	32.10 ± 15.11	33.76 ± 10.23	41.17 ± 12.56	.000
Time before peak (s)	0.52 ± 0.32	0.46 ± 0.17	0.38 ± 0.22	.000
Time after peak (s)	0.70 ± 0.59	0.62 ± 0.16	0.52 ± 0.25	.006
Tonic contractions				
Average peak amplitude (uV)	36.57 ± 17.65	39.75 ± 15.28	45.31 ± 27.34	.016
Average mean amplitude (uV)	21.85 ± 12.05	26.77 ± 6.17	29.71 ± 6.56	.000
Mean amplitude variability (%)	0.26 ± 0.12	0.23 ± 0.08	0.20 ± 0.06	.000
Endurance contractions				
Average mean amplitude (uV)	18.85 ± 10.56	25.70 ± 8.62	32.33 ± 10.58	.000
Mean amplitude variability (%)	0.22 ± 0.09	0.21 ± 0.07	0.19 ± 0.05	.001
Rest Post-baseline				
Average mean amplitude (uV)	4.86 ± 3.36	4.31 ± 3.49	3.48 ± 1.85	.008
Mean amplitude variability (%)	0.25 ± 0.27	0.24 ± 0.21	0.19 ± 0.10	.085

### 3.4. *Comparison of the* PFM *function at 102 days postpartum*

Upon reexamining the PFM function at 102 days postpartum, we found that the evaluation values of group A in the rest pre-baseline stage, the time before and after peak of phasic (flick) contractions stage, and the endurance contractions stage were significantly improved as than those in group B (*P *< .05), as displayed in Table 4.

**Table 4:** Comparison of the each value of pelvic floor sEMG assessed by Glazer at 102 days postpartum.

	Group A (n = 105)	Group B (n = 93)	*P*
Rest pre-baseline			
Average mean amplitude (uV)	2.72 ± 2.08	3.93 ± 2.70	.000
Mean amplitude variability (%)	0.20 ± 0.11	0.22 ± 0.19	.314
Phasic (flick) contractions			
Average peak amplitude (uV)	42.14 ± 15.51	41.17 ± 12.56	.628
Time before peak (s)	0.30 ± 0.09	0.38 ± 0.22	.002
Time after peak (s)	0.44 ± 0.13	0.52 ± 0.25	.008
Tonic contractions			
Average peak amplitude (uV)	45.65 ± 12.35	45.31 ± 27.34	.907
Average mean amplitude (uV)	32.18 ± 10.99	29.71 ± 6.56	.061
Mean amplitude variability (%)	0.19 ± 0.05	0.20 ± 0.06	.307
Endurance contractions			
Average mean amplitude (uV)	28.78 ± 13.01	32.33 ± 10.58	.036
Mean amplitude variability (%)	0.20 ± 0.06	0.19 ± 0.05	.025
Rest Post-baseline			
Average mean amplitude (uV)	3.89 ± 9.12	3.48 ± 1.85	.652
Mean amplitude variability (%)	0.20 ± 0.09	0.19 ± 0.10	.234

*Group A: Biofeedback plus electrical stimulation; Group B: Kegel exercise.

### 3.5. *Differences in the* PFM *function in different delivery modes*

The pelvic floor EMG values of different delivery modes were compared. We found that on day 42 after parturition, each index of the tonic contractions stage was higher after spontaneous labor than that after cesarean; the differences were all significant, *P* < .05, but on day 102 postpartum, the pelvic floor EMG values were the same irrespective of the delivery mode (Table 5).

**Table 5:** Glazer evaluation of pelvic floor EMG in different delivery modes at different time after delivery.

		42th			72th			102th		
		Cesarean	Eutocia	*P*	Cesarean	Eutocia	*P*	Cesarean	Eutocia	*P*
Rest pre-baseline	Average Mean Amplitude(uV)	10.45 ± 13.74	7.66 ± 10.65	.112	6.01 ± 11.53	4.98 ± 7.33	.456	3.45 ± 2.37	3.12 ± 2.26	.342
	Mean Amplitude Variability (%)	0.39 ± 0.39	0.40 ± 0.53	.081	0.27 ± 0.23	0.30 ± 0.25	.441	0.19 ± 0.11	0.22 ± 0.19	.157
Phasic (flick) contractions	Average Peak Amplitude (uV)	37.36 ± 16.92	30.56 ± 15.10	.003	36.59 ± 10.53	33.43 ± 10.39	.035	43.041 ± 3.32	40.29 ± 14.94	.173
	Time Before Peak (s)	0.50 ± 0.26	0.57 ± 0.37	.121	0.43 ± 0.18	0.44 ± 0.19	.629	0.34 ± 0.22	0.33 ± 0.10	.561
	Time After Peak (s)	0.62 ± 0.51	0.78 ± 0.69	.072	0.53 ± 0.24	0.58 ± 0.33	.258	0.48 ± 0.21	0.48 ± 0.19	.850
Tonic contractions	Average Peak Amplitude (uV)	34.25 ± 15.87	29.80 ± 12.71	.031	39.13 ± 13.77	38.53 ± 14.34	.766	47.32 ± 23.12	43.63 ± 17.89	.210
	Average Mean Amplitude (uV)	25.39 ± 12.20	20.70 ± 12.38	.008	28.61 ± 9.00	28.07 ± 11.29	.710	30.77 ± 7.78	31.28 ± 10.56	.701
	Mean Amplitude Variability (%)	0.24 ± 0.09	0.29 ± 0.14	.013	0.21 ± 0.07	0.22 ± 0.75	.774	0.20 ± 0.06	0.19 ± 0.06	.427
Endurance contractions	Average Mean Amplitude (uV)	22.31 ± 12.13	17.23 ± 10.19	.002	26.66 ± 8.51	23.83 ± 8.46	.020	30.96 ± 10.36	29.92 ± 13.56	.547
	Mean Amplitude Variability (%)	0.22 ± 0.08	0.23 ± 0.09	.281	0.20 ± 0.08	0.21 ± 0.74	.520	0.20 ± 0.06	0.20 ± 0.05	.919
Rest Post-baseline	Average Mean Amplitude (uV)	5.40 ± 3.32	4.47 ± 2.56	.290	3.80 ± 2.95	3.85 ± 2.59	.881	4.14 ± 9.37	3.25 ± 1.60	.351
	Mean Amplitude Variability (%)	0.29 ± 0.41	0.25 ± 0.30	.440	0.25 ± 0.18	0.24 ± 0.19	.786	0.19 ± 0.09	0.20 ± 0.10	.264

### 3.6. *Differences in the* PFM *function in women of different ages*

The results of the analysis of the postpartum PFM function of women of different ages are presented in Table 6. This table displays that only in the phasic (flick) contractions stage on day 42 were the values for the younger mothers obviously higher, *P *= .025. The other stage values for mothers of different ages during different time periods were not statistically significant, *P* > .05.

**Table 6:** Glazer evaluation of pelvic floor EMG in different ages at different time after deliver.

		42th			72th			102th		
		20–30 (y)	31–40 (y)	*P*	20–30 (y)	31–40 (y)	*P*	20–30 (y)	31–40 (y)	*P*
Rest pre-baseline	Average Mean Amplitude (uV)	8.44 ± 10.72	9.36 ± 13.07	.602	6.55 ± 13.15	5.01 ± 7.56	.297	2.96 ± 2.26	3.44 ± 2.55	.180
	Mean Amplitude Variability (%)	0.43 ± 0.47	0.38 ± 0.46	.481	0.22 ± 0.16	0.31 ± 0.26	.017	0.23 ± 0.22	0.20 ± 0.11	.207
Phasic (flick) contractions	Average Peak Amplitude (uV)	38.06 ± 18.07	32.10 ± 15.21	.025	34.85 ± 9.93	35.12 ± 10.87	.864	44.83 ± 14.422	40.21 ± 13.87	.360
	Time Before Peak (s)	0.53 ± 0.28	0.53 ± 0.33	.892	0.41 ± 0.15	0.44 ± 0.20	.153	0.33 ± 0.09	0.34 ± 0.20	.445
	Time After Peak (s)	0.66 ± 0.44	0.72 ± 0.68	.497	0.51 ± 0.25	0.58 ± 0.30	.103	0.50 ± 0.19	0.470.20	.267
Tonic contractions	Average Peak Amplitude (uV)	33.75 ± 16.72	31.25 ± 13.37	.301	38.55 ± 12.47	38.97 ± 14.73	.837	50.26 ± 26.71	43.27 ± 16.91	.060
	Average Mean Amplitude (uV)	25.17 ± 13.71	22.09 ± 11.79	.126	30.36 ± 10.80	27.40 ± 9.77	.067	30.34 ± 6.41	31.3410.31	.480
	Mean Amplitude Variability (%)	0.29 ± 0.15	0.26 ± 0.10	.090	0.20 ± 0.76	0.22 ± 0.07	.176	0.20 ± 0.51	0.19 ± 0.60	.904
Endurance contractions	Average Mean Amplitude (uV)	21.26 ± 12.65	19.11 ± 10.85	.247	26.57 ± 8.57	24.64 ± 8.55	.142	29.56 ± 10.10	30.86 ± 12.85	.442
	Mean Amplitude Variability (%)	0.23 ± 0.08	0.23 ± 0.09	.826	0.22 ± 0.08	0.22 ± 0.08	.940	0.20 ± 0.05	0.20 ± 0.06	.949
Rest Post-baseline	Average Mean Amplitude (uV)	4.82 ± 3.10	4.99 ± 2.96	.708	4.19 ± 2.65	3.65 ± 2.82	.193	4.55 ± 11.68	3.30 ± 1.83	.401
	Mean Amplitude Variability (%)	0.28 ± 0.48	0.26 ± 0.25	.759	0.26 ± 0.22	0.24 ± 0.17	.448	0.21 ± 0.10	0.19 ± 0.09	.293

## 4. Discussion

PFD disease is a group of illnesses due to pelvic floor support structure defects, damage, and dysfunction, thus resulting in pelvic organ displacement and leading to various pelvic organ dysfunctions.^[17]^ It is 1 of the 5 common chronic diseases threatening women’s sexual health after hypertension, depression, diabetes, and osteoporosis.^[7,18]^

PFD is known as social cancer^[19]^ and mainly includes urinary incontinence, POP, and fecal incontinence. In developing countries, the incidence rate of these 3 diseases is 28.7%, 19.7%, and 6.9%,^[20]^ respectively. The risk factors include parity, vaginal delivery, aging, and obesity.^[14]^ An epidemiological survey of urinary incontinence in China demonstrated that delivery is the independent and first risk factor leading to PFM injury and PFD.^[21]^ Valeton^[22]^ considered the delivery time to be a risk factor for PFM injury, which is consistent with Wesnes’s research.^[11]^ During pregnancy, the incidence of urinary incontinence in multipara is 1.32 times that in primipara and 1.05 times after childbirth.^[11]^ However, after reasonable pelvic floor rehabilitation exercise and treatment, PFD caused by pregnancy and childbirth can be restored reversibly.

### 4.1. Biofeedback adds electrical stimulation therapy

Biofeedback can amplify, process, and transform physiological signals that people have not noticed into perceptible signals through a PFM biofeedback therapy instrument, allowing patients to correctly adjust or control their internal organs or body functions according to these signals to treat certain diseases. Pelvic floor electrical stimulation uses electrical stimulation technology to excite the corresponding innervated nerves and muscles, which can enhance the contractile ability of the PFMs and prevent PFM injury and atrophy. Castro^[23]^ demonstrated that after 8 weeks of electrical stimulation, urinary incontinence improved by 50% and was almost completely cured after 12 weeks. Parkkinen^[24]^ also found that electrical stimulation combined with PFM exercise for 12 months demonstrated a 97% improvement in symptoms. Electrical stimulation and/or biofeedback can improve the motivation and compliance to treatment for women who cannot actively contract the PFM.^[25]^

This study revealed that only after 1 month of biofeedback plus electrical stimulation therapy, the symptoms of urinary retention, constipation, urinary incontinence, fecal incontinence, and POP were notably improved. Two months later, the function of the PFM was almost normal, and the effect of the electrical stimulation was better than that of the Kegel training in terms of improving urine retention, but its specific efficacy and long-term effects need to be studied further.

### 4.2. PFM *training*

In 1948, Arnold Kegel first proposed this campaign for postpartum female urinary incontinence, pointing out that these exercises help to prevent cystocele, rectocele, and stress urinary incontinence.^[26]^ A meta-analysis also depicted that the symptoms and severity of patients with mild PFD can be alleviated and improved through PFM rehabilitation training and that such training can delay the disease progression.^[14]^ The National Institute for Health and Clinical Excellence also recommended PFM training for at least 3 months as the preferred conservative treatment for patients with stress urinary incontinence (class A evidence).^[27]^

In this study, the Kegel training group began to exercise daily from day 42 after delivery. After 1 month of training, the PFM evaluation demonstrated that the pelvic floor surface EMG was almost better than that before exercise, but there was no statistical significance. After 2 months of training, the evaluation results revealed that the symptoms of urinary incontinence, fecal incontinence, and sexual dysfunction were dramatically improved. Therefore, short-term exercise cannot fully demonstrate its efficacy of this exercise. Cavkaytar suggested that 8 weeks is the shortest time to strengthen pelvic muscles,^[28]^ while National Institute for Health and Clinical Excellence suggested that the shortest time is 3 months.^[27]^ Hence, we will continue to provide sufficient information and guidance to this group of pregnant women and carry out regular Kegel exercise sessions to further demonstrate the long-term effectiveness of Kegel exercise.

Previous studies have considered vaginal delivery is a risk factor for PFD; however, the results of this study depict that only in the early postpartum period, the pelvic floor function of vaginal delivery was worse than that of the cesarean section. With the extension of the postpartum time, there was no significant difference in the pelvic floor function among the different delivery modes. We also found that in mothers under 40 years old, the pelvic floor function was unrelated to age. Considering the social and economic development, people’s living standards have improved, and nutrition is sufficient. Therefore, women’s understanding of the PFM gradually increased, and they began to pay attention to pelvic floor rehabilitation and other factors. Hence, this study believes that now vaginal delivery is unnecessarily more likely to aggravate PFM injury than a cesarean section in the long term, but this still needs more in-depth research.

This study excluded the pelvic floor EMG test and the follow-up data of postpartum women without PFM rehabilitation training and could thus not evaluate the natural outcome of the PFM of untreated second-birth women. Moreover, there was no long-term comparison between the untreated and the rehabilitation training groups. We will conduct a more detailed study later.

In short, all women, irrespective of whether they have PFD symptoms after the first delivery, should start and insist on PFM exercise under the correct guidance of medical staff 1 week or even earlier after postpartum in combination with their own conditions.^[10]^

## Author contributions

**Conceptualization:** Yan Shen.

**Data curation:** Jun Chao Zeng, Yu Ying Yang.

**Formal analysis:** Jun Chao Zeng.

**Investigation:** Jun Chao Zeng, Yan Shen.

**Methodology:** Yu Ying Yang, Yan Shen.

**Resources:** Yu Ying Yang, Yan Shen.

**Software:** Yu Ying Yang.

**Writing – original draft:** Yan Shen.
